# Supplementary material for: HIVprotI: an integrated web based platform for prediction and design of HIV proteins inhibitors
Source: J Cheminform. 2018 Mar 9;10:12. doi: 10.1186/s13321-018-0266-y (PMC5845081; doi:10.1186/s13321-018-0266-y)
Supplement: Supplementary file 2 — Additional file 2. Source code of HIVProtI web server. [file 13321_2018_266_MOESM2_ESM.zip › HIVprotI_Source-code/hivprotI/weka-3-6-6/doc/weka/classifiers/functions/supportVector/RBFKernel.html]

RBFKernel


---


|  |  |  |
| --- | --- | --- |
| **Overview** \| **Package** \| **Class** \| **Tree** \| **Deprecated** \| **Index** \| **Help** \| **Weka's home** | |  |
| **PREV CLASS**   **NEXT CLASS** | **FRAMES**    **NO FRAMES**     **All Classes** |  |
| SUMMARY: NESTED \| FIELD \| CONSTR \| METHOD | DETAIL: FIELD \| CONSTR \| METHOD |  |


---


## weka.classifiers.functions.supportVector Class RBFKernel

```
java.lang.Object
  weka.classifiers.functions.supportVector.Kernel
      weka.classifiers.functions.supportVector.CachedKernel
          weka.classifiers.functions.supportVector.RBFKernel
```

**All Implemented Interfaces:**: java.io.Serializable, CapabilitiesHandler, OptionHandler, RevisionHandler

**Direct Known Subclasses:**: MIRBFKernel

---

``` public class RBFKernel extends CachedKernel ```

The RBF kernel. K(x, y) = e^-(gamma \* <x-y, x-y>^2)


Valid options are:

```
 -D
  Enables debugging output (if available) to be printed.
  (default: off)
```

```
 -no-checks
  Turns off all checks - use with caution!
  (default: checks on)
```

```
 -C <num>
  The size of the cache (a prime number), 0 for full cache and 
  -1 to turn it off.
  (default: 250007)
```

```
 -G <num>
  The Gamma parameter.
  (default: 0.01)
```

**Version:**
:   $Revision: 5518 $

**Author:**
:   Eibe Frank (eibe@cs.waikato.ac.nz), Shane Legg (shane@intelligenesis.net) (sparse vector code), Stuart Inglis (stuart@reeltwo.com) (sparse vector code), J. Lindgren (jtlindgr{at}cs.helsinki.fi) (RBF kernel)

**See Also:**: Serialized Form

---

**Constructor Summary**

`RBFKernel()`
  
          default constructor - does nothing.

`RBFKernel(Instances data,
int cacheSize,
double gamma)`
  
          Constructor.


| **Method Summary** | |
| --- | --- |
| `void` | `buildKernel(Instances data)`             builds the kernel with the given data. |
| `java.lang.String` | `gammaTipText()`             Returns the tip text for this property |
| `Capabilities` | `getCapabilities()`             Returns the Capabilities of this kernel. |
| `double` | `getGamma()`             Gets the gamma value. |
| `java.lang.String[]` | `getOptions()`             Gets the current settings of the Kernel. |
| `java.lang.String` | `getRevision()`             Returns the revision string. |
| `java.lang.String` | `globalInfo()`             Returns a string describing the kernel |
| `java.util.Enumeration` | `listOptions()`             Returns an enumeration describing the available options. |
| `void` | `setGamma(double value)`             Sets the gamma value. |
| `void` | `setOptions(java.lang.String[] options)`             Parses a given list of options. |
| `java.lang.String` | `toString()`             returns a string representation for the Kernel |

**Methods inherited from class weka.classifiers.functions.supportVector.CachedKernel**

`cacheSizeTipText, clean, eval, getCacheSize, numCacheHits, numEvals, setCacheSize`

**Methods inherited from class weka.classifiers.functions.supportVector.Kernel**

`checksTurnedOffTipText, debugTipText, forName, getChecksTurnedOff, getDebug, makeCopies, makeCopy, setChecksTurnedOff, setDebug`

**Methods inherited from class java.lang.Object**

`equals, getClass, hashCode, notify, notifyAll, wait, wait, wait`

**Constructor Detail**

### RBFKernel

```
public RBFKernel()
```

:   default constructor - does nothing.

---


### RBFKernel

```
public RBFKernel(Instances data,
                 int cacheSize,
                 double gamma)
          throws java.lang.Exception
```

:   Constructor. Initializes m\_kernelPrecalc[].

    **Parameters:**: `data` - the data to use: `cacheSize` - the size of the cache: `gamma` - the bandwidth **Throws:**: `java.lang.Exception` - if something goes wrong


**Method Detail**

### globalInfo

```
public java.lang.String globalInfo()
```

:   Returns a string describing the kernel

    :   **Specified by:**: `globalInfo` in class `Kernel`
    :   **Returns:**: a description suitable for displaying in the explorer/experimenter gui

---


### listOptions

```
public java.util.Enumeration listOptions()
```

:   Returns an enumeration describing the available options.

    :   **Specified by:**: `listOptions` in interface `OptionHandler` **Overrides:**: `listOptions` in class `CachedKernel`
    :   **Returns:**: an enumeration of all the available options.

---


### setOptions

```
public void setOptions(java.lang.String[] options)
                throws java.lang.Exception
```

:   Parses a given list of options.


    Valid options are:

    ```
     -D
      Enables debugging output (if available) to be printed.
      (default: off)
    ```

    ```
     -no-checks
      Turns off all checks - use with caution!
      (default: checks on)
    ```

    ```
     -C <num>
      The size of the cache (a prime number), 0 for full cache and 
      -1 to turn it off.
      (default: 250007)
    ```

    ```
     -G <num>
      The Gamma parameter.
      (default: 0.01)
    ```

    :   **Specified by:**: `setOptions` in interface `OptionHandler` **Overrides:**: `setOptions` in class `CachedKernel`
    :   **Parameters:**: `options` - the list of options as an array of strings **Throws:**: `java.lang.Exception` - if an option is not supported

---


### getOptions

```
public java.lang.String[] getOptions()
```

:   Gets the current settings of the Kernel.

    :   **Specified by:**: `getOptions` in interface `OptionHandler` **Overrides:**: `getOptions` in class `CachedKernel`
    :   **Returns:**: an array of strings suitable for passing to setOptions

---


### setGamma

```
public void setGamma(double value)
```

:   Sets the gamma value.

    :   **Parameters:**: `value` - the gamma value

---


### getGamma

```
public double getGamma()
```

:   Gets the gamma value.

    :   **Returns:**: the gamma value

---


### gammaTipText

```
public java.lang.String gammaTipText()
```

:   Returns the tip text for this property

    :   **Returns:**: tip text for this property suitable for displaying in the explorer/experimenter gui

---


### getCapabilities

```
public Capabilities getCapabilities()
```

:   Returns the Capabilities of this kernel.

    :   **Specified by:**: `getCapabilities` in interface `CapabilitiesHandler` **Overrides:**: `getCapabilities` in class `Kernel`
    :   **Returns:**: the capabilities of this object **See Also:**: `Capabilities`

---


### buildKernel

```
public void buildKernel(Instances data)
                 throws java.lang.Exception
```

:   builds the kernel with the given data. Initializes the kernel cache.
    The actual size of the cache in bytes is (64 \* cacheSize).

    :   **Overrides:**: `buildKernel` in class `CachedKernel`
    :   **Parameters:**: `data` - the data to base the kernel on **Throws:**: `java.lang.Exception` - if something goes wrong

---


### toString

```
public java.lang.String toString()
```

:   returns a string representation for the Kernel

    :   **Overrides:**: `toString` in class `java.lang.Object`
    :   **Returns:**: a string representaiton of the kernel

---


### getRevision

```
public java.lang.String getRevision()
```

:   Returns the revision string.

    :   **Specified by:**: `getRevision` in interface `RevisionHandler` **Overrides:**: `getRevision` in class `Kernel`
    :   **Returns:**: the revision


---


|  |  |  |
| --- | --- | --- |
| **Overview** \| **Package** \| **Class** \| **Tree** \| **Deprecated** \| **Index** \| **Help** \| **Weka's home** | |  |
| **PREV CLASS**   **NEXT CLASS** | **FRAMES**    **NO FRAMES**     **All Classes** |  |
| SUMMARY: NESTED \| FIELD \| CONSTR \| METHOD | DETAIL: FIELD \| CONSTR \| METHOD |  |


---
